# Secreted Factors from Human Vestibular Schwannomas Can Cause Cochlear Damage

**DOI:** 10.1038/srep18599

**Published:** 2015-12-22

**Authors:** Sonam Dilwali, Lukas D. Landegger, Vitor Y. R. Soares, Daniel G. Deschler, Konstantina M. Stankovic

**Affiliations:** 1 Eaton Peabody Laboratories, Massachusetts Eye and Ear Infirmary, 243 Charles Street, Boston, MA 02114, USA; 2 Department of Otolaryngology, Massachusetts Eye and Ear Infirmary, 243 Charles Street, Boston, MA 02114, USA; 3 Harvard-MIT Program in Health, Science and Technology, 77 Massachusetts Avenue, Cambridge, MA 02139, USA; 4 Department of Otorhinolaryngology-Head and Neck Surgery, Medical University of Vienna, Waehringer Guertel 18-20, 1090 Vienna, Austria; 5 Department of Otology and Laryngology, Harvard Medical School, 25 Shattuck St, Boston, MA 02115, USA; 6 Department of Otorhinolaryngology-Head and Neck Surgery, Health Science Faculty, University of Brasilia, SGAN, Via L2 Norte, Quadra 604/605, 70840-050, Asa Norte, DF, Brazil

## Abstract

Vestibular schwannomas (VSs) are the most common tumours of the cerebellopontine angle. Ninety-five percent of people with VS present with sensorineural hearing loss (SNHL); the mechanism of this SNHL is currently unknown. To establish the first model to study the role of VS-secreted factors in causing SNHL, murine cochlear explant cultures were treated with human tumour secretions from thirteen different unilateral, sporadic VSs of subjects demonstrating varied degrees of ipsilateral SNHL. The extent of cochlear explant damage due to secretion application roughly correlated with the subjects’ degree of SNHL. Secretions from tumours associated with most substantial SNHL resulted in most significant hair cell loss and neuronal fibre disorganization. Secretions from VSs associated with good hearing or from healthy human nerves led to either no effect or solely fibre disorganization. Our results are the first to demonstrate that secreted factors from VSs can lead to cochlear damage. Further, we identified tumour necrosis factor alpha (TNFα) as an ototoxic molecule and fibroblast growth factor 2 (FGF2) as an otoprotective molecule in VS secretions. Antibody-mediated TNFα neutralization in VS secretions partially prevented hair cell loss due to the secretions. Taken together, we have identified a new mechanism responsible for SNHL due to VSs.

Vestibular schwannomas (VSs) are the most common tumours of the cerebellopontine angle and the fourth most common intracranial neoplasms. Although VSs arise from the vestibular nerves, 95% of patients with VS present with sensorineural hearing loss (SNHL). The underlying pathophysiological mechanisms of this SNHL are currently unknown^1^.

Vestibular schwannomas occur sporadically or in association with neurofibromatosis type II (NF2), a debilitating disease whose hallmark is bilateral VSs. In addition to SNHL, VSs can cause facial paralysis, disequilibrium, other cranial neuropathies and even death from brainstem compression^2^.

Currently, there are no FDA approved drugs to prevent or treat VS or the associated SNHL. However, two classes of drugs have demonstrated some efficacy in ameliorating SNHL due to VS via unknown mechanisms: bevacizumab, a monoclonal antibody against vascular endothelial growth factor A (VEGF-A) improves hearing in 54% of NF2-associated VS^3^, whereas corticosteroids can improve sudden SNHL associated with sporadic and NF2-associated VS^4^. These clinical clues and the unmet medical need to prevent and treat VS-associated SNHL motivate our work in understanding the mechanism of SNHL due to VS.

The predominant hypothesis has been that VSs cause SNHL by mechanically compressing the adjacent auditory nerve. However, this hypothesis does not explain the lack of correlation between the radiographic tumour size or tumour extent within the internal auditory canal and audiometric threshold shifts in people with sporadic VS^5^,^6^. Further, some patients develop audiometric threshold shifts despite the lack of VS growth^6^.

The loss or damage of structures within the inner ear due to VS has been implicated in previous work. Sound-induced vibration of fluids within the inner ear leads to stimulation of cochlear sensory hair cells and excitation of the auditory nerve, which induces activity in more central auditory centres. In addition to behavioural threshold audiometry, there are two commonly used physiologic metrics for evaluation of auditory function: distortion-product otoacoustic emissions (DPOAEs), which are generated by cochlear outer hair cells (OHCs); and auditory brainstem evoked response (ABR), which is a surface potential consisting of multiple waves, with wave I representing the summed activity of the cochlear nerve, and later waves representing contributions from ascending auditory nuclei in the brainstem. Although VSs lead to delayed propagation and decreased amplitude of ABR neural impulses^7^,^8^, they also reduce DPOAE amplitudes, consistent with cochlear dysfunction. Importantly, decreased DPOAEs are present in VS patients with mild SNHL^9^, suggesting that OHC dysfunction could be primary, happening early in the progression of the SNHL, rather than secondary to auditory nerve fibres or neurons. These physiologic findings *in vivo* are corroborated by *post mortem* histopathological analyses of temporal bones of individuals with untreated VS-specifically, substantial ipsilateral cochlear atrophy, including degeneration of organ of Corti that comprises sensory hair cells, loss of spiral ganglion neurons, and atrophy of the stria vascularis^10^. However, no published work has elucidated whether the cochlear, arising within the inner ear, or retrocochlear dysfunction, originating centrally to the inner ear, precedes the other.

An alternative to mechanical compression of the auditory nerve leading to SNHL due to VS is the hypothesis, initially explored by our laboratory, that there are biological differences between VSs that cause SNHL and those that do not^11^. Using cDNA microarrays, we found that VS stratified by hearing can have different gene expression profiles, suggesting that diverging concentrations of potentially ototoxic or otoprotective molecules may contribute to the degree of SNHL seen in VS patients^11^. A subsequent cDNA microarray study discovered that PDGF-A gene expression levels inversely correlated with SNHL in VS patients^12^, thus supporting our view that intrinsic genetic differences in VSs could contribute to SNHL.

Radiologic studies have suggested that differences in cochlear fluids contribute to SNHL associated with VS. Specifically, the differential intensity of fluid-attenuated inversion recovery magnetic resonance imaging (MRI) sequences from the cochlea, most likely representing the protein density in cochlear fluids, correlates with the degree of SNHL due to NF2-associated and sporadic VSs^13^,^14^,^15^. Importantly, unlike sporadic VS, NF2-associated VS size correlates with the degree of SNHL, suggesting that mechanical compression may be an important factor in SNHL due to NF2 VSs^16^, which is why our work focuses on sporadic VS-associated SNHL.

We explore potential ototoxic or neurotoxic biological secretions from VSs that could reach the cochlea via the fundus of the internal auditory canal so to alter cochlear function and hearing. We have previously shown that the proteome of cochlear fluid is different in patients with VS versus without VS^17^, suggesting a role of VS-secreted factors in cochlear cell health. Our proteomic study was motivated by earlier reports that total levels of protein in perilymph are 5–15 times higher than in healthy individuals, a difference that was used to diagnose VS prior to the advent of MRI^13^,^14^. However, no published work thus far has shown a direct effect of VS-associated molecules in causing inner ear degeneration.

Here, we assessed damage in mouse cochlear explants subjected to human VS secretions. Based on the results in the histopathological study of human temporal bones affected by VS^10^, we focused on hair cell and neurite loss, while testing VS secretions obtained from subjects with different degrees of ipsilateral SNHL. Our findings suggest that there is a correlation between degree of SNHL in human VS patients and damage caused by the application of tumour secretions on murine cochlear explants. This is the first demonstration that the VS-secreted factors can directly lead to cochlear damage. We have further identified correlations between two secreted molecules and the degree of SNHL, with tumour necrosis factor alpha (TNFα) demonstrating an ototoxic and fibroblast growth factor 2 (FGF2) an otoprotective potential.

## Results

### Patient Demographics

**Figure 1:**
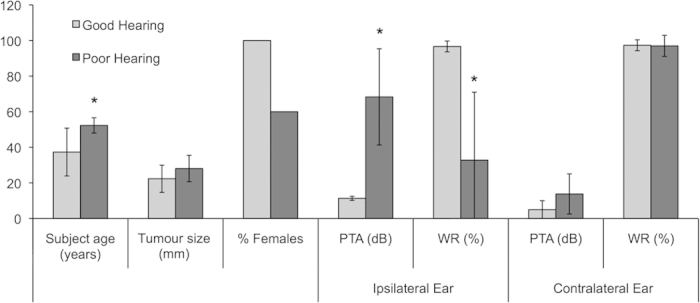
Patient demographics for VS secretions applied to cochlear explants. Average subject age in years, tumour size (mm, largest transverse dimension), % females, pure tone average (PTA, dB), and word recognition score (WR, %) are given for the ipsilateral and contralateral ears to VS. Data are segregated into groups of VS associated with good hearing (PTA < 30 dB and WR > 70%, n = 3 being VS2, VS5 and VS9) and poor hearing (n = 10). * = p < 0.05.

Demographics of patients with unilateral, sporadic VSs whose tumours were utilized to collect secretions are summarized in Fig. 1 and Table 1. The contralateral ear did not have significant SNHL in any of the patients except for VS7 and VS13. Out of the thirteen patients, three had good hearing (GH) in the ipsilateral ear (VS2, VS5 and VS9). Age of subject (p = 0.007) and ipsilateral pure tone average (PTA) (p = 0.005) were significantly greater, whereas ipsilateral word recognition score (WR) (p = 0.017) was significantly lower in poor hearing subjects.

### Loss of hair cells and neurites in cochlear explants due to vestibular schwannoma secretions

**Figure 2:**
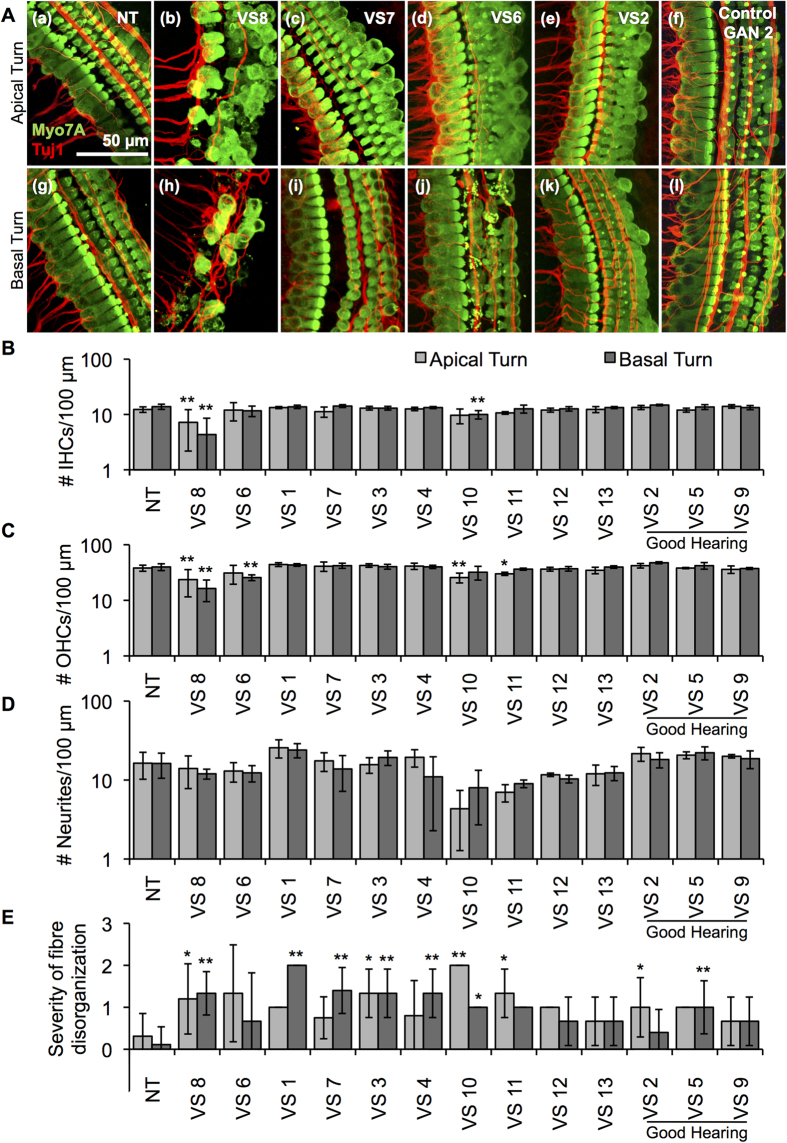
Application of human VS secretions onto murine cochlear explant cultures leads to hair cell and neurite loss. (A). Representative images for cochlear explants receiving (a) no treatment (NT, n = 28 different explants), incubated with (b) VS8 (n = 5 different explants), (c) VS7 (n = 4 different explants), (d) VS6 (n = 3 different explants), (e) VS2 (n = 5 different explants) and (f) control GAN2 (n = 3 different explants) secretions are shown for the apical turns, and (g) NT (n = 26 different explants), (h) VS8 (n = 6 different explants), (i) VS7 (n = 5 different explants), (j) VS6 (n = 3 different explants), (k) VS2 (n = 5 different explants) and (l) control GAN2 (n = 4 different explants) secretions for the basal turn. Myo7A (green) marks hair cells and Tuj1 (red) marks neurites. Scale Bar = 50 μm applies to all images. (**B**). Number of inner hair cells (IHCs), (**C**). outer hair cells (OHCs), (**D**). neurites, and (**E**). severity of fibre disorganization are shown for a 100 μm length within the apex (light grey columns) and basal turn (dark grey columns) cochlear explants treated with NT and secretions from 13 different tumours. *p < 0.05, **p < 0.01. Quantified data after treatment with control GAN secretions are shown in Supplementary Figure 1.

Application of VS secretions, collected by incubating fresh sporadic VS specimens in culture medium for 72 hours, onto neonatal murine cochlear explants led to variable types and degree of damage in the cochlear cells. Control healthy nerve secretions were collected from surgically sacrificed healthy human great auricular nerves (GANs) in the same manner. We hypothesized that VS secretions from tumours associated with GH or control GAN secretions would not lead to significant cochlear damage, whereas VS secretions from tumours associated with poor hearing (PH) would cause cochlear damage. As anticipated, no hair cell or neurite loss was noted due to secretions from three control GANs or VSs associated with GH (VS2, VS5 and VS9) when specific types of cochlear cells were counted along a 100 μm length in confocal microscopy images. The only significant morphological change was disorganization of the nerve fibres, assessed as a qualitative measure with 0 being highly organized and 2 being severely disorganized, for secretions from two GH tumours (VS2 and VS5) and from one GAN (GAN1). Further, VS secretions from tumours associated with PH (VS1, VS3, VS4, VS6-VS8 and VS10-13) led to a much greater degree of damage in the cochlear explants, exhibiting significant loss of IHCs or OHCs, or fibre disorganization. Apical and basal turns of the murine cochleae were cultured as separate cochlear explants; a greater degree of damage was noted in the basal turns overall. Representative projection images are shown for cochlear explants treated with control media (no treatment (NT)), VS8, VS7, VS6 and VS2 secretions in Fig. 2A (apical turn a–e, basal turn g–k), respectively. Representative images are also shown for cochlear explants treated with GAN secretions (GAN2) in Fig. 2A (apical turn f, basal turn l).

Quantification of confocal images is summarized as average ± standard deviation (SD). N represents the number of different cochlear explant cultures tested for a given treatment. Benjamini-Hochberg adjusted t-test p-values are reported. Data from the cochlear apex and base are analyzed separately because they correspond to different frequency regions. IHC counts per 100 μm length showed VS8 secretions (from a tumour associated with anacusis) leading to significant IHC loss at the apical and basal turns; number of IHCs decreased from 12.3 ± 1.4 (n = 28) for NT to 7.2 ± 5.0 (n = 5, p = 0.0005) with treatment in the apex, and from 13.9 ± 1.5 (n = 26) for NT to 4.3 ± 4.2 (n = 6, p = 10^−9^) with treatment in the base (Fig. 2B). Additionally, treatment of explants with VS10 secretions (another tumour associated with anacusis) led to significant IHC loss at the basal turn; number of IHCs decreased to 10 ± 1.7 (n = 3, p = 0.002). Treatment of explants with control GAN secretions did not significantly change IHC counts in the apex or base (Supplementary Fig. 1). N is the same in the rest of analyses as for IHC counts for all treatments.

Four of the ten poor hearing tumours caused OHC loss. VS8, VS10 and VS11 secretions led to significant OHC loss in the apical turn: the number of OHCs per 100 μm length decreased from 38.1 ± 4.8 for NT to 23.6 ± 12.1 (p = 0.0004) for VS8, to 25.7 ± 5.1 (p = 0.001) for VS10 and 30.0 ± 2.0 (p = 0.03) for VS11 (Fig. 2C). For the basal turn, VS6 and VS8 secretions led to significant OHC loss, decreasing from 39.8 ± 5.6 for NT to 25.7 ± 3.1 (p = 0.001) and 16.3 ± 6.9 (p = 10^−8^), respectively (Fig. 2C). Application of control GAN secretions did not significantly change OHC counts in the apex or base (Supplementary Fig. 1).

Although VS9, VS10 and VS11 secretions showed a trend toward reduced neurites with application, the trends did not meet significance (Fig. 2D). The number of neurites per 100 μm length did not significantly change in the apical or basal turns after application of control GAN secretions (Supplementary Fig. 1).

The severity of nerve fibre disorganization was assessed qualitatively with 0 standing for essentially intact and 2 being most severe. The radial fibres arising from the modiolar region consist of the majority of afferents from the IHCs, with the rest being efferents. The spiraling fibres in the periphery of the cochlear explant are thought to mostly contain efferents coming from the medial olivary complex within the superior olive that synapse onto OHCs to modulate their function as amplifiers, with a smaller population being afferents^18^. Although more proximal centers, such as the superior olive, are not present in the cochlear explant cultures, nerve fibres synapsing at the IHCs and OHCs remained intact and organized in control specimens (Fig. 2A(a)).

Interestingly, nerve fibre disorganization was noted in explants treated with secretions. In the apical turn, secretions from VS2, VS3, VS8, VS10 and VS11 led to significant fibre disorganization, all tumours being associated with PH except VS2. Specifically, severity of fibre disorganization per 100 μm length for the apical turn decreased from 0.3 ± 0.5 for NT group to 1.3 ± 0.6 (p = 0.01), 1.2 ± 0.8 (p = 0.01), 2.0 ± 0.0 (p = 0.008) and 1.3 ± 0.6 (p = 0.03) for explants treated with VS3, VS8, VS10 and VS11 secretions, respectively (Fig. 2E). For VS2, level of fibre disorganization was 1.0 ± 0.7 (p = 0.04), the least drastic change out of all the significant changes. In the basal turn, secretions from VS1, VS3, VS4, VS5, VS7, VS8 and VS10 led to significantly increased fibre disorganization; only VS5 is associated with GH. The values were 0.1 ± 0.5 for NT, 2.0 ± 0.0 (p = 10^−8^) for VS1, 1.3 ± 0.6 (p = 0.004) for VS3, 1.3 ± 0.6 (p = 0.0004) for VS4, 1.0 ± 0.6 (p = 0.0008) for VS5, 1.4 ± 0.5 (p = 10^−5^) for VS7, 1.3 ± 0.5 (p = 10^−5^) for VS8, and 1.0 ± 0.0 (p = 0.05) for VS10 (Fig. 2E). The severity of neurite fibre disorganization was not affected by GAN secretions, except for secretions from GAN1 in the apical turn only, increasing from 0.1 ± 0.4 for NT to 1.0 ± 0.0 (p = 0.04) with GAN1 secretion treatment (Supplementary Fig. 1).

Overall, differential levels of damage were noted due to different VS secretions. There was a general trend of increasing damage from apical to basal turns. Osmolality for the randomly selected tumour secretions did not significantly deviate from control media, being 323, 316 and 324 mOsm/kg for control media, VS7 and VS8 secretions, respectively. The values were near the expected value of 335 mOsm/kg in perilymph for mammals^19^.

### TNFα correlates directly and FGF2 correlates inversely with degree of SNHL due to VS

**Figure 3:**
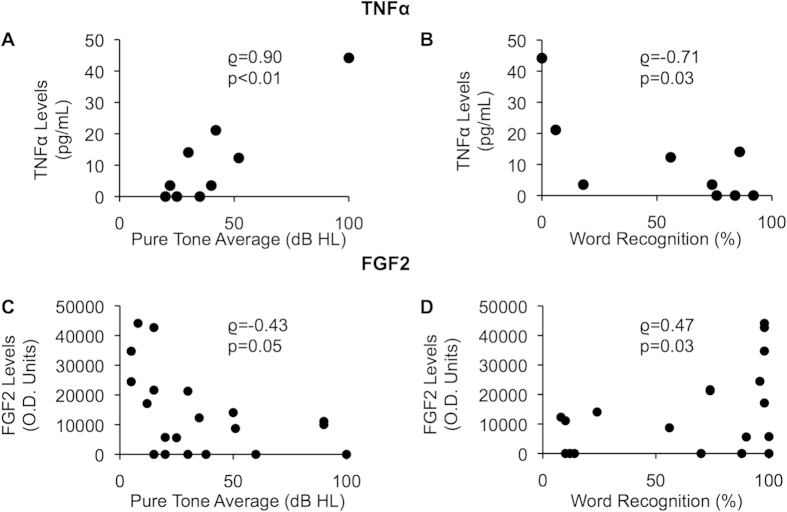
Correlation of VS-secreted TNFα and FGF2 with SNHL due to VS. (**A**). Correlation of pure tone average (dB) and (**B**). word recognition score (%) of ipsilateral ear to the VS, and measured secreted TNFα levels from VSs (n = 9). (**C**). Correlation of pure tone average (dB) and (**D**). word recognition score (%) of ipsilateral ear to the VS and measured secreted FGF2 levels from VSs (n = 21). p-values are shown, rho represents Spearman’s rank correlation coefficient.

TNFα concentrations were measured in nine tumour secretions, collected in phosphate buffered saline (PBS), using ELISA. These sporadic VS secretions had to be obtained from different tumours than the VS secretions used for the explant experiments described above because of the small specimen size. We focused on TNFα because it is a pro-inflammatory molecule involved in many diseases, including SNHL^20^, and the therapeutic effect of anti-inflammatory corticosteroids in treating sudden SNHL due to VS suggests a pathogenic role of inflammatory molecules in SNHL due to VS. The patients whose VS secretions were used had varied levels of ipsilateral SNHL, with pure tone average ranging from 20–100 dB and word recognition score ranging from 0–92%. The contralateral ears for all patients had normal hearing. TNFα levels, on average being 11.0 pg/mL, had a range of 0.0 to 44.2 pg/mL. TNFα levels in the secretions correlated positively and significantly with the subjects’ pure tone averages (rho = 0.90, p = 0.0009, Fig. 3A), and correlated negatively with the word recognition scores (rho = −0.71, p = 0.03, Fig. 3B) in the ipsilateral ear.

Reanalyzing cytokine array data that we have previously published^21^ for FGF2 levels in 21 VS secretions collected in PBS, FGF2 expression correlated negatively with the pure tone average (rho = −0.43, p = 0.05, Fig. 3C), and correlated positively with word recognition score (rho = 0.47, p = 0.03, Fig. 3D) in the ipsilateral ear as the VS.

### Tumour necrosis factor alpha application alone leads to loss and disorganization of neurites in basal turn of cochlear explants

**Figure 4:**
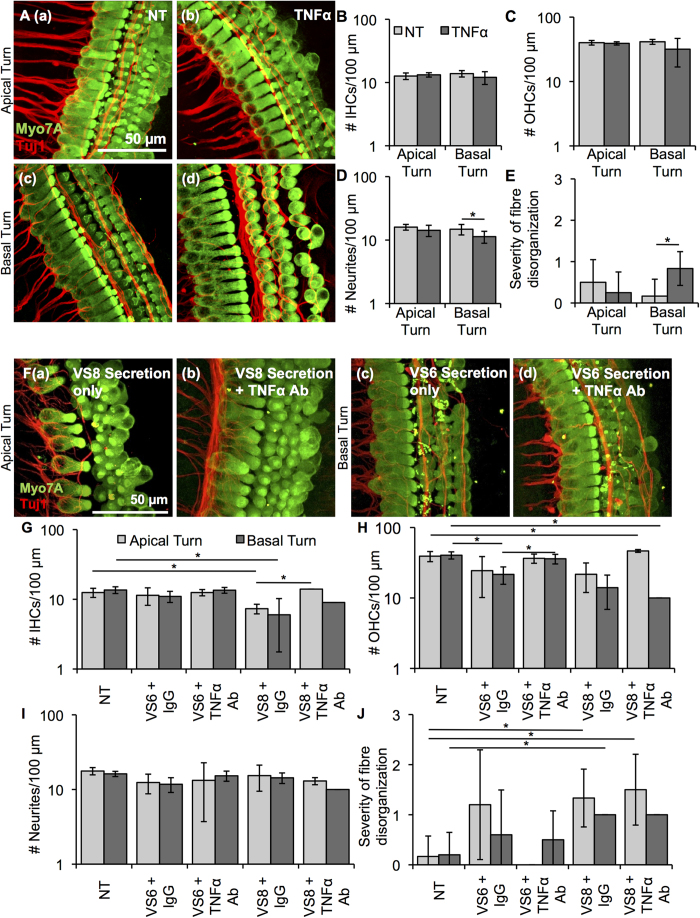
TNFα application onto cochlear explants induced mild damage. (**A**). Representative images for cochlear explants receiving no treatment (NT, a, c, n = 6 different explants) or incubated with TNFα (b, d, n = 4–6 different explants) are shown for the apical (a, b) and basal (c, d) turn. Myo7A (green) marks hair cells and Tuj1 (red) marks neurites. Scale Bar = 50 μm applies to all images. (**B**). Number of inner hair cells (IHCs), (**C**). number of outer hair cells (OHCs), (**D**). number of neurites, (**E**). severity of fibre disorganization are shown for a 100 μm length within the apical and basal turn explants for NT (light grey columns) and TNFα-treated (dark grey columns). *p < 0.05. (**F**). Representative images for cochlear explants receiving VS secretions only (a, c, n = 2 different tumours) or incubated with VS secretions with TNFα antibody (b, d, n = 2 different tumours) are shown for the apical (a, b) and basal (c, d) turn. Myo7A (green) marks hair cells and Tuj1 (red) marks neurites. Scale Bar = 50 μm applies to all images. (**G**). Number of inner hair cells (IHCs), (**H**). number of outer hair cells (OHCs), (**I**). number of neurites, (**J**). severity of fibre disorganization are shown for a 100 μm length within the apical and basal turn explants treated with VS secretions only (light grey columns) and VS secretions with TNFα antibody (dark grey columns). *p < 0.05.

To understand if tumour necrosis factor alpha (TNFα) has the potential of causing SNHL, we applied recombinant TNFα onto cochlear explant cultures. Cell counts and morphology of recombinant human TNFα-treated cochlear explants were compared with those of age-matched control specimens receiving only PBS (NT). Specific damage in terms of neurite counts and fibre organization of the treated samples was detected in the basal turn. Representative images for NT and TNFα-treated cultures are shown in Fig. 4A (apical turn a–b, basal turn c–d), respectively. Data are summarized as average ± SD for NT and TNFα-treated cochlear explants. N represents the number of different cochlear explant cultures tested for a given treatment. The number of IHCs (Fig. 4B) or OHCs (Fig. 4C) per 100 μm length along the cochlea did not change in the apical or basal turn. While the number of OHCs tended to decrease in the basal turn, the change did not meet significance. Although the neurite counts did not change significantly in the apical turn, they did change significantly in the basal turn, going from 14.8 ± 2.8 for NT (n = 6) to 11.3 ± 2.4 with treatment (n = 6, p = 0.04) (Fig. 4D). The severity of the fibre disorganization, assessed qualitatively with 0 being essentially intact and 2 being most severe, did not change significantly in the apical turn, but did change significantly in the basal turn, going from 0.2 ± 0.4 for NT (n = 6) to 0.8 ± 0.4 with treatment (n = 6, p = 0.02) (Fig. 4E). Osmolality did not deviate from control media, being 330 and 329 mOSm/kg for control media and media with recombinant TNFα, respectively.

### TNFα neutralization in VS secretions partially prevents hair cell loss due to VS secretions in cochlear explants

In order to more directly assess TNFα’s role in VS secretions leading to cochlear damage, secretions from two tumours that caused significant cochlear explant damage and secreted substantial levels of TNFα based on ELISA, namely VS6 and VS8, were incubated with a goat anti-human TNFα antibody prior to application onto cochlear explants. After 2 h of incubation of the secretions with a TNFα antibody or control goat IgG antibody, the secretions, along with antibody, were applied to the cochlear explants for 48 h following the same protocol as for applying secretions alone. Antibody-mediated TNFα neutralization was successful in reducing TNFα levels to 3.7 pg/mL from 17.6 pg/mL in VS6 secretions as measured using ELISA. Interestingly, TNFα neutralization could prevent hair cell loss for VS6 and VS8 (Fig. 4 (F–J)). Specifically, there was significant loss of IHCs due to VS8 in both apical and basal turns, decreasing IHCs from 12.5 ± 1.8 (n = 6) and 13.6 ± 1.5 (n = 5) for NT to 7.3 ± 1.5 (p = 0.03, n = 3) and 6.0 ± 4.2 (p = 0.03, n = 4) for apical and basal turns, respectively (Fig. 4G). TNFα neutralization could significantly prevent the apical IHC loss, increasing the number of hair cells per 100 μm to 14.0 ± 0.0 (p = 0.02, n = 2) (Fig. 4G). Further, OHC loss caused by VS6 in the basal turn, decreasing from 40.4 ± 4.7 (n = 5) to 21.6 ± 5.9 (p = 0.03, n = 5) could be prevented by TNFα neutralization, increasing the number of hair cells per 100 μm to 36.0 ± 5.6 (p = 0.03, n = 4) (Fig. 4H). The number of neurites did not significantly change with secretion application or TNFα neutralization (Fig. 4I). Although severity of fibre disorganization was significantly increased with VS8 secretion application, it could not be prevented by TNFα neutralization (Fig. 4J). Our results suggest TNFα as a key player in causing hearing loss due to VS and that neutralization of TNFα should be explored as a preventive therapy against VS-induced SNHL.

### Recombinant VEGF application leads to decreased HGF secretion without notable morphological changes in cochlear explants

**Figure 5:**
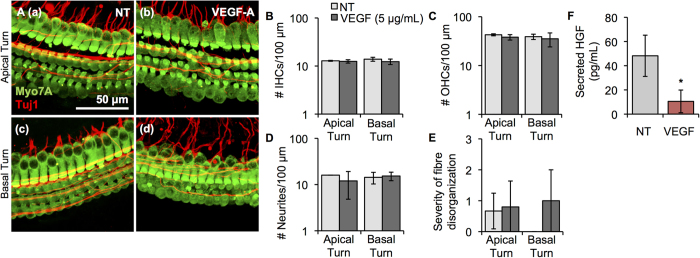
VEGF-A application onto cochlear explants did not induce substantial cellular damage but did significantly decrease HGF secretion. (**A**). Representative images for cochlear explants receiving no treatment (NT, a, c, n = 3–4 different explants) or incubated with VEGF-A (b, d, n = 3–5 different explants) are shown for the apical (a, b) and basal (c, d) turn. Myo7A (green) marks hair cells and Tuj1 (red) marks neurites. Scale Bar = 50 μm applies to all images. (**B**). Number of inner hair cells (IHCs), (**C**). number of outer hair cells (OHCs), (**D**). number of neurites, (**E**). severity of fibre disorganization are shown for a 100 μm length within the apical and basal turn explants for NT (light grey columns) and VEGF-A-treated (dark grey columns) (**F**). Secreted murine HGF levels in cochlear explants post-VEGF treatment. *p < 0.05.

Administering bevacizumab, a VEGF monoclonal antibody, to patients with NF2-associated VSs results in hearing improvement independent of tumour shrinkage^3^,^22^. The mechanism behind this improvement in hearing is currently unknown. To understand the role of VEGF in causing SNHL due to VS, we applied recombinant VEGF onto cochlear explants. No significant loss of IHCs (Fig. 5B), OHCs (Fig. 5C), neurites (Fig. 5D) or fibre organization (Fig. 5E) was noted in the apical (n = 5) and basal turns (n = 3) when compared to NT controls (n = 4). However, there was a trend for a decreased number of OHCs in the apical and basal turns, and a trend for a decreased number of neurites in the apical turn. Representative projection images of control apical and basal turns and VEGF-A-treated apical and basal turns are shown in Fig. 5A (a–d respectively). Osmolality did not deviate from control media, being 330 mOSm/kg for both control media and media with recombinant VEGF-A.

Previous work from our laboratory demonstrating cross-talk between VEGF and hepatocyte growth factor (HGF) in primary human vestibular schwannoma and Schwann cells^23^ motivated us to explore cross-talk between VEGF-A and HGF in cochlear explants as a potential mechanism through which VEGF-A can cause SNHL due to VS. HGF is an interesting molecule because its levels in the cochlea are tightly regulated: too much or too little HGF causes SNHL^24^. To understand VEGF-A and HGF’s relationship in cochlear cells, HGF levels were measured after VEGF-A addition. Basal secreted HGF levels were 48.1 ± 17.0 pg/mL in control cochlear explants (n = 4, Fig. 5F). Treating cochlear explants with 5 μg/mL recombinant human VEGF-A for 48 hours led to significantly lower levels of HGF, being 10.5 ± 9.4 pg/mL (n = 4, p = 0.01, Fig. 5F). This change was specific to VEGF-A since incubating the cochlear explants with the same concentration of TNFα did not lead to changes in the secreted HGF levels (n = 3, p = 0.40).

## Discussion

We show, for the first time, that VS-secreted factors can lead to cochlear degeneration. Our study was motivated by previous work that demonstrates the lack of correlation between the radiographic tumour size or tumour extent within the internal auditory canal and audiometric threshold shifts in people with sporadic VS^5^,^6^. Our results support our hypothesis that secreted factors from VS can lead to oto- and neurotoxicity. While application of solely culture medium or secretions from healthy GANs led to no detectable cochlear damage (with the exception of mild fibre disorganization noted in response to secretions from only 1 control nerve), secretions from different tumours resulted in variable patterns of cochlear damage *in vitro*, providing mechanistic insight into the variable degree of cochlear histopathology observed *post mortem* in people with VS^5^. Overall, the damage noted in the cochlear explants after VS secretion application was reflective of the patient’s degree of ipsilateral SNHL. For instance, VS8, from a patient with a deaf ear, led to drastic degeneration of the cochlea, including loss of HCs and neurites, changes that would cause profound SNHL, with increasing severity from the apical to basal turn as commonly noted in VS patients^1^.

In contrast to VS secretions from a patient with ipsilateral anacusis, many other VS secretions caused more specific damage: secretions from VS6, removed from a subject with a moderate SNHL, demonstrated specific loss of OHCs in the basal turn, potentially explaining the elevated pure tone average of 40 dB, as OHC dysfunction is thought to lead to 40–60 dB threshold shift in hearing^25^. The patient’s mild decrease in word recognition on the ipsilateral side suggests potential dysfunction of the neural pathway along with HC loss, a pathology that could be explained through neurite loss noted in the cochlear explant cultures.

Interestingly, although VS7 and VS8 patients presented with an essentially deaf ear, their secretions led to very different changes in the cochlear explants, with VS7 secretions causing primarily fibre disorganization. There could be several reasons for this difference. First, VS7 secretions’ induction of substantial fibre disorganization could have led to the audiometric results noted (≥100 dB pure tone average and 0% word recognition) even with the rest of the cochlear structures being intact. If the fibres are disorganized and dysfunctional, then the information is not transmitted from the HCs to the more central auditory centres. Indeed, synaptic loss and disorganization, without loss of HCs or neurons, has been described in other pathologies characterized by SNHL, including Meniere’s disease^26^. Clinically, if DPOAE testing, which reflects outer hair cell function, was available in the patient with VS7, we would have expected it to be normal despite profound deafness, as observed in people with auditory neuropathy^27^. Second, as the patient with VS7 also had a history of exposure to bilateral loud noise, and moderate SNHL contralaterally (34 dB pure tone average, 84% word recognition), the ipsilateral ear could have been more vulnerable to VS than if the ear had not been acoustically traumatized. Third, as we hypothesize that the SNHL due to VS is multifactorial, different VSs could cause SNHL in different ways^1^. The main mechanism behind the SNHL due to VS7 could be more so nerve compression or tumour-associated oedema rather than secreted factors, whereas for VS8, it could be mainly due to the secreted factors. However, auditory nerve compression by VS7 is unlikely to be the main mechanism of SNHL because VS7 was substantially smaller than VS8 *in vivo*, and the underlying assumption is that extent of compression is correlated with tumour size. Other than the tumour’s physical size, there could be other factors influencing VS-mediated SNHL. In our study, three of the younger subjects had the best pure-tone hearing, albeit another young patient suffered from anacusis (tumour VS10). Subjects with good hearing were significantly younger than patients with poor hearing (Fig. 1). Age and gender effects on VS secretions have not been studied in the past. Nonetheless, age of patients has been found to correlate with level of hearing loss^28^. In another study, females with VS seem to have a lower incidence of hearing loss (HL) and smaller tumours than males^29^. It could be possible that age or gender of the subject as well as age of the tumour are influencing the profile of secreted proteins from these tumours. Future work is warranted to investigate the age and gender effects in order to gain a better understanding of the factors influencing these tumours’ ototoxic and neurotoxic ability.

To delineate these various possibilities, our findings strongly motivate future clinical studies that would entail serial audiometric testing, including DPOAE testing, combined with serial collection of perilymph, CSF and serum in VS patients.

In the meantime, our model and hypotheses are strengthened by the results that secretions from all three VS associated with GH or GANs did not lead to significant HC or neurite loss in the cochlear explants. Interestingly, VS2 and VS5, tumours associated with GH, did cause significant fibre disorganization in the only apical turn or basal turn, respectively. It may be that the putative damage due to VS5 secretions *in vivo* was in the basal region encoding frequencies higher than the highest frequency tested by threshold audiometry. Early, asymptomatic SNHL can be detected when hearing is tested above the frequency range evaluated regularly (<8 kHz)^30^. It may be possible that secretions from healthy nerves or GH tumours do not lead to significant fibre disorganization *in vivo* due to counteracting homeostatic mechanisms that maintain fibre organization, a feature that was lacking in our *in vitro* model. Nonetheless, the lack of cochlear cell loss due to application of secretions from VS of subjects with GH or GAN secretions provides confidence that the degeneration seen is due to the specific molecules present in the VS secretions, rather than due to the paradigm.

Using a candidate molecule approach, we focused on two proteins that may be orchestrating SNHL due to VS: TNFα as a putative ototoxic molecule, and FGF2 as a putative otoprotective molecule. We found, for the first time, the robust correlation between TNFα levels in VS secretions and degree of SNHL, in terms of both pure tone average and word recognition. This result is intriguing because TNFα is known to cause SNHL in other aetiologies, based on elevated TNFα serum levels in people with idiopathic sudden SNHL^31^ and immune-mediated SNHL^32^. Similar to VS secretions causing more severe damage in the basal than apical turn, TNFα-induced damage in IHCs and nerve fibres specifically in the basal turn. Applying TNFα onto the cochlear explants did not induce severe damage as has been described previously when applying TNFα at the same concentration (1 μg/mL) to rat cochlear explants^33^. This is most likely because we did not use the lowest most part of the basal turn near the hook region, where Dinh *et al.* noted most of the damage^33^. We also cultured the cochlear explants intact with neuronal connections that could provide growth factors and protective molecules, whereas Dinh *et al.* cultured only the organ of Corti. Differences could also be due to variability in species susceptibility (mouse versus rat), because we used TNFα from a different species (human) than the derived cochlear explants (mouse), or because of differences in source and purity of recombinant TNFα compared to previous studies. Nonetheless, our study is the first to identify an ototoxic secreted molecule in the context of VS. Probing TNFα’s precise role in VS-mediated SNHL, we assessed whether antibody-mediated TNFα neutralization could prevent cochlear explant damage noted with secretion application. Intriguingly, TNFα neutralization could prevent IHC loss due to one tumour’s secretions (VS8) and prevent OHC loss (VS6) in another case. It is possible that TNFα has differing ototoxic effects based on a given tumour’s secretome or other properties of the tumour. TNFα inhibition via monoclonal antibodies has been clinically successful in alleviating immune-mediated and idiopathic SNHL^34^, and our study suggests that it could also be beneficial for VS-mediated SNHL.

In contrast to the positive correlation between TNFα levels in VS secretions and degree of SNHL, we found a negative correlation between FGF2 levels in VS secretions and degree of SNHL. This is interesting because FGF2 is a known otoprotective molecule in other contexts^35^,^36^. Although we previously reported on this correlation for FGF2, when analyzing the *average* levels of FGF2 in VS secretions associated with good versus PH^21^, we now provide additional insight into the strength of this correlation by plotting *individual* FGF2 levels from VS secretions and comparing them with pure tone average and word recognition from the same subject. Taken together, our data suggest that subjects whose VS secrete high levels of FGF2 and low levels of TNFα will most likely retain their hearing even while having the VS. Our results motivate future development of a prognostic test for SNHL due to VS, possibly based on serum or CSF levels of TNFα and FGF2.

Based on the therapeutic efficacy and unknown mechanism of anti-VEGF-A antibody, bevacizumab, in rescuing SNHL in some NF2 patients with VS^3^, we explored whether exogenous VEGF-A application to cochlear explants could be toxic. We found no morphologic damage of cochlear cells exposed to exogenous VEGF-A. Interestingly, we did find that VEGF-A application specifically decreased the explants’ HGF secretion. This is significant because human mutations in HGF are associated with nonsyndromic SNHL^24^, while mice that lack or overexpress HGF are deaf^24^, indicating that cochlear HGF levels need to be carefully controlled to assure normal hearing. Increased cochlear VEGF-A expression has been described in various cell types, including spiral ganglion neurons and the stria vascularis, after different inner ear insults^37^, including after noise trauma^38^ and vibration-induced SNHL in guinea pigs^39^. Importantly, application of recombinant HGF to cochlear explant cultures has been shown to significantly reduce the HC loss induced by aminoglycosides, and local application of HGF to the round window membrane of guinea pigs attenuates noise-induced HL^40^. Therefore, increasing HGF levels by VEGF-A inhibition could be otoprotective and potentially be the mechanism of hearing improvement with bevacizumab in NF2 VS patients. It also may be that VEGF-A is acting along with several other molecules, such as TNFα or FGF2, to create a cumulative ototoxicity profile due to VS. Further, limitations of our model system such as usage of immature cochlear explants and the ability of only short-term assessment could have produced divergent results *in vitro* versus what is noted *in vivo* although others have demonstrated the utility of the neonatal cochlear explant system for screening of drugs that also worked in the mature cochlea *in vivo*^41^,^42^.

We demonstrate, for the first time, direct cochlear damage due to VS-secreted factors. Tumour secretions led to variable types of damage, including loss of hair cells and neurites, and neurite disorganization; the damage tended to increase in severity from apical to the basal turn, and correlated with the severity of SNHL. These results with cochlear explants provide mechanistic insight into the variable damage of cochlear cells noted in *post mortem* histopathologic analyses of human temporal bones with untreated VS, where cochlear damage was much more severe in VS associated with poor than with GH^10^. We identified VS-secreted TNFα and FGF2 as some of the first ototoxic and otoprotective molecules, respectively, which may be modulating SNHL due to VS. Our results support the hypothesis that VS-secreted factors can damage or protect cochlear cells, controlling the SNHL in VS patients.

## Methods

### Study population and human specimen collection

Surgical VS specimens were collected from patients with sporadic VSs. Control great auricular nerves (GANs) were obtained from patients undergoing neck dissections or parotidectomies, during which these nerves are routinely sacrificed. Informed consent was obtained from all patients. The study protocols were approved by the Human Studies Committee of Massachusetts General Hospital and Massachusetts Eye and Ear Infirmary, and conducted in accordance with the Helsinki Declaration. We utilized GAN as the control for the following reasons: (1) GAN is a sensory nerve like the vestibular nerves with a robust sheath of Schwann cells; (2) tumours developing on this nerve are exceptionally rare (we could not find any schwannomas on the GAN described in the literature), making it unlikely that the tissue we utilized had neoplastic properties; (3) healthy GANs are routinely sacrificed during neck surgery for access to deeper neck structures so we have ready access to control tissue, (4) the cochlear nerve that is sometimes sacrificed during resection of the adjacent VS cannot be assumed to be “healthy” because it shares the tumour microenvironment with the VS, and it may harbor pre-tumourous molecular changes as it is anatomically contiguous with the vestibular nerves at the root entry zone of CNVIII. Specimens were placed in sterile saline on ice for 15 minutes while being transported to the laboratory. Age was defined at the time of diagnosis. Tumour size (largest diameter parallel to the petrous face), PTA (the average of the lowest thresholds (in dB) for two tones among 0.5, 1 and 2 kHz) and WR (the percentage of spoken words a subject can comprehend) were from the last measurements prior to tumour surgery. A deaf ear was assigned a PTA of 100 dB and WR of 0%.

### Preparation of vestibular schwannoma secretions

After separating cauterized and hemorrhagic parts of the fresh sporadic VS or GAN specimens, the sample was rinsed with sterile PBS thrice. VS and GAN secretions were collected by incubating a washed, fresh tumour or control specimen in 100% DMEM for 3 days at 37°C and 5% CO_2_ levels in sterile conditions. The secretions were normalized by weight (0.1 g specimen/0.1 mL DMEM). In addition, DMEM alone was incubated in parallel as control media (used for no treatment (NT)). Following the removal of the tumour piece, secretions were frozen at −80°C until further use.

### Murine cochlear explant culture

To develop an animal model to study SNHL due to VS, cochlear explant cultures were established from CBA/CaJ mice (Jackson Laboratory, ME) on postnatal day 3–5. The study protocol was approved by the Institutional Animal Care and Use Committee of Massachusetts Eye and Ear Infirmary. Briefly, after decapitation, temporal bones were surgically separated from the skull in Hank’s Balanced Salt Solution (Life Technologies, NY). The bony otic capsule encasing the cochlea was dissected away. The spiral ligament was carefully stripped away along the cochlear length, starting from the base, to expose the organ of Corti with sensory hair cells connected to the spiral ganglion neuron region. The lower apical and upper basal turns were microdissected separately, providing two pieces per cochlea. The upper apical turn and hook of the basal turn were discarded as they were often damaged during dissection. For brevity, the lower apical and upper basal turns are referred to as “apical” and “basal” in the remainder of manuscript. Reissner’s membrane was removed. The explants were cultured onto 10 mm glass coverslips coated with Cell-Tak (BD Biosciences, CA, #354241) in a 35 mm culture dish with 4 wells. Culture medium consisted of 98% DMEM, 1% ampicillin and 1% N2 supplement.

### Treatment of cochlear explant cultures with vestibular schwannoma or healthy nerve secretions

After approximately 12 hours of culturing, the cochlear explant cultures were treated for 48 hours with tumour or control healthy nerve (without schwannomas) secretions diluted to half their concentration. The exact formulation was 46.5% tumour- or nerve-conditioned DMEM, 46.5% plain DMEM, 5% FBS, 1% ampicillin and 1% N2 supplement. All reagents were purchased from Life Technologies, NY. Importantly, for any experimental treatment, control explants receiving no treatment (NT, composed of 46.5% DMEM incubated without specimen for 3 days simultaneously as DMEM incubated with specimen, 46.5% plain DMEM, 5% FBS, 1% ampicillin and 1% N2 supplement)) from age-matched littermates were analyzed in parallel.

In order to understand TNFα’s effect on cochlear damage due to VS secretions, TNFα neutralization was conducted in two tumour secretions (VS6 and VS8) by incubating secretions in a goat anti-TNFα antibody (R&D Systems) for 2 h prior to application onto cochlear explants. TNFα neutralization was assessed in VS6 secretions after incubation with anti-TNFα antibody through ELISA (R&D Systems). Control experiments encompassed incubating NT media with anti-TNFα antibody, or incubating VS secretions or NT media with goat IgGs using the same protocol. After incubation with TNFα antibodies or goat IgGs, VS secretions were applied along with the antibody to cochlear explants for 48 h before fixation for immunofluorescence and imaging.

### Measurements of secretions

Osmolality in VS secretions was measured using a vapor-pressure osmometer (standardized with 290 mOsm/kg of 0.1567M sodium chloride solution) before applying them onto cochlear explants.

Extra formulation for some VS secretions (collected either in PBS for 1 hour or in DMEM for 3 days) was made to be tested on ELISA. Human TNFα and FGF2 ELISAs (R&D Systems) were conducted as directed by the manufacturer on VS secretions prior to being applied onto the cochlear explant cultures. Mouse HGF ELISA kit was purchased from R&D systems and manufacturer’s protocols were followed.

### Treatment of cochlear explant cultures with recombinant TNFα and VEGF

Recombinant human TNFα or human VEGF (R&D Systems), diluted in culture media to a 5 μg/mL concentration, was applied to cochlear explants for 48 hours. Control cochlear explants received the same volume of PBS in the media as needed to add recombinant TNFα. The culture media was saved for analysis after treatment and the explants were fixed for immunofluorescence.

### Immunofluorescence and imaging

After treatment, cochlear explants were rinsed in phosphate-buffered saline (PBS), fixed with 4% paraformaldehyde (Electron Microscopy Sciences, PA) in PBS for 20 minutes, washed with PBS, treated for 5 minutes, exposed to a blocking buffer consisting of 5% Normal Horse Serum (NHS, Sigma-Aldrich, MO) with 1% Triton-X (Integra Chemical, WA) for 1 hour, and incubated with antibodies against myosin 7A (Myo7A, Proteus Biosciences, CA, 1:200) and β-tubulin (Tuj1, Covance, MA, 1:200) diluted in 1% NHS with 1% Triton-X overnight at 4 °C, to identify hair cells (HCs) and neurons, respectively. After PBS washes, secondary antibodies (Alexa Fluor 555 anti-mouse and Alexa Fluor 488 anti-rabbit, Life Technologies, 1:1000) diluted in 1% NHS with 1% Triton-X were applied for 2 hours. Nuclear staining was performed with one 2-minute immersion in Hoechst stain 33342 (Life Technologies, NY, 1 nM dilution) followed by two 5-minute PBS washes. The coverslips were mounted on glass slides using Vectashield (Vector Laboratories, CA, #H-1000). The edges of the coverslips were sealed with clear nail polish (Electron Microscopy Sciences, PA). Cochlear explants were imaged using a Leica TCS SP5 Confocal microscope. Zoomed-in pictures for the region of organ of Corti, including neurites, were merged in a z-stack to obtain a z-axis projection image in the Leica software. The number of inner (IHCs) and outer hair cells (OHCs) and neurites were manually counted per 100 μm length along the cochlea, with 1–2 samplings per specimen. The 100 μm section for analysis was used for all analyses as it was deemed a long enough length for an accurate count but a short enough length for it to be consistently evaluated between specimens and has been commonly used in previous literature (e.g.^41^). The neurites were counted and assessed approximately 10 μm beneath the inner hair cells. Fibre organization, including both afferent radial neurites from the IHCs and efferent spiralling neurites to the OHCs, was assessed using a qualitative scale with 0 being essentially intact and 2 equalling severe disorganization. Supplementary Figure 2 illustrates the differences between a specimen assigned severities of 0,1 and 2 and highlights two regions within the image where neurite disorganization was assessed, i.e. the afferent radial neurites and the efferent spiraling neurites. Disorganization was defined as being 0 if the majority of the neurites were aligned perpendicularly to the hair cells’ horizontal axis, were easily distinguishable from each other and were not very tortuous. Disorganization was defined as being 1 if the neurites were not aligned perpendicularly to the hair cells’ horizontal axis, neurites were tangled with other neighboring neurites and were more tortuous. Disorganization was defined as being 2 if the neurites had a beading or tortuous appearance (i.e. the path of the neurites to the hair cells was not apparent), were not aligned with each other or perpendicularly aligned to the hair cells, and the ends of the neurites generally did not terminate at the basal parts of the hair cells. All assessments and analyses were conducted in a blinded-fashion.

### Statistical analyses

For the cytokine array, proteins were determined to be significantly expressed if the corresponding spots had optical densities more than 2 standard deviations of background values above the mean background level for each array. For the cytokine array and ELISA, statistical significance was determined through an analysis of variance (ANOVA) with alpha set to 0.05. For all cochlear explant experiments, differences were analyzed using a two-tailed t-test. For application of secretions, Benjamini-Hochberg correction for multiple hypotheses was conducted on generated p-values and Benjamini-Hochberg corrected p-values ≤ 0.05 were considered significant.

## Additional Information

**How to cite this article**: Dilwali, S. *et al.* Secreted Factors from Human Vestibular Schwannomas Can Cause Cochlear Damage. *Sci. Rep.* **5**, 18599; doi: 10.1038/srep18599 (2015).

## Supplementary Material

Supplementary Information

## Figures and Tables

**Table 1:**
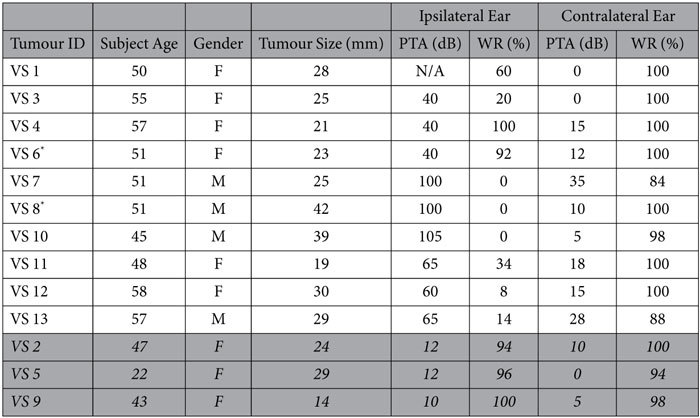
Subject demographics for VS secretions applied to cochlear explants for 13 tumours.

Subject age, gender, tumour size (mm, largest transverse dimension), pure tone average (PTA, dB), and word recognition score (WR, %) are given for the ipsilateral and contralateral ears to VS. The rows shaded in grey are for VSs associated with GH. N/A = not available.^*^In an alternate series of experiments, these tumour secretions were subjected to TNF neutralization prior to application.
