# Supplementary material for: Secreted Factors from Human Vestibular Schwannomas Can Cause Cochlear Damage
Source: Sci Rep. 2015 Dec 22;5:18599. doi: 10.1038/srep18599 (PMC4686978; doi:10.1038/srep18599)
Supplement: Supplementary Information [file srep18599-s1.pdf]

Supplementary Figures

Manuscript Title: Secreted Factors from Human Vestibular Schwannomas Can Cause Cochlear Damage

Authors: Sonam Dilwali, Lukas D. Landegger,
Vitor Y.R. Soares, Daniel G. Deschler
and Konstantina M. Stankovic

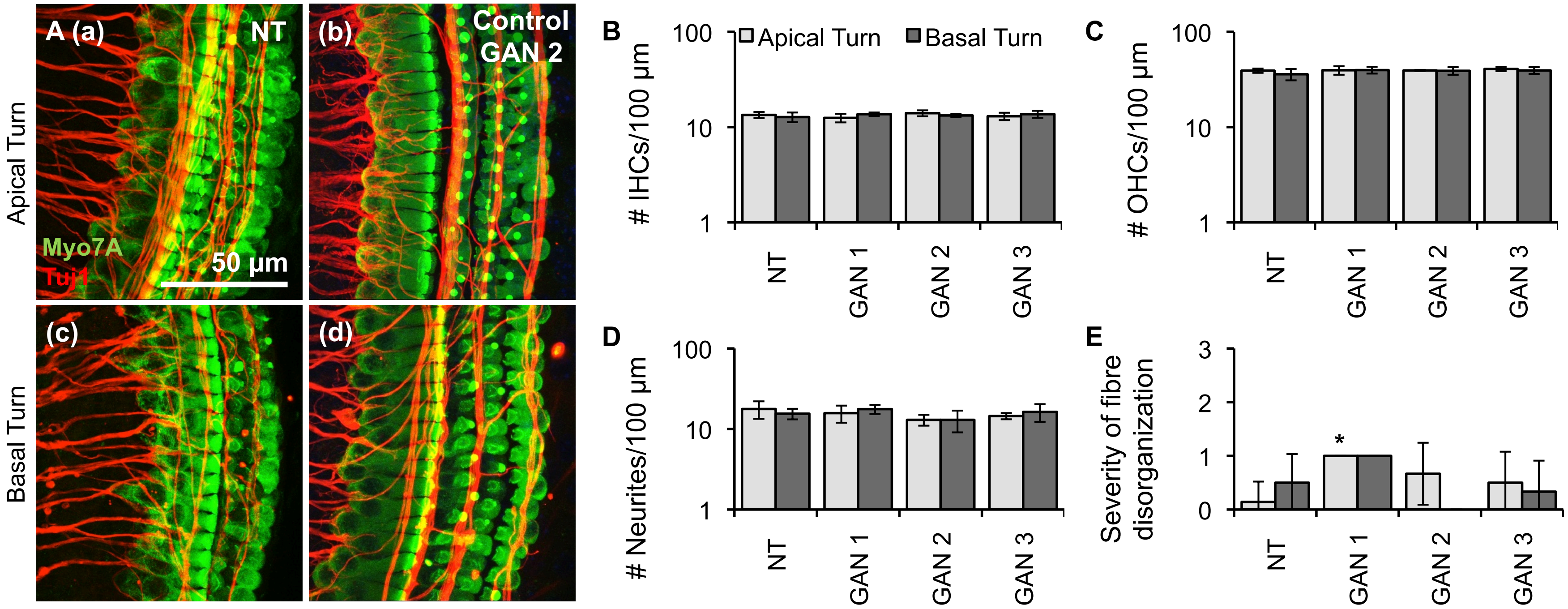

Supplementary Figure 1. Application of human GAN secretions onto murine cochlear explant cultures does not lead to hair cell and neurite loss. **A**. Representative images for cochlear explants receiving **(a)** no treatment (NT, n=7 different explants) or with **(b)** GAN2 (n=3 different explants) secretions are shown for the apical turn, and **(c)** NT (n=8 different explants) or **(d)** GAN2 (n=4 different explants) secretions for the basal turn. Myo7A (green) marks hair cells and Tuj1 (red) marks neurites. Scale Bar = 50 μm applies to all images. **B.** Number of inner hair cells (IHCs), **C.** outer hair cells (OHCs), **D.** neurites, and **E.** severity of fibre disorganization are shown for a 100 μm length within the apex (light grey columns) and basal turn (dark grey columns) cochlear explants treated with NT and secretions from 3 different GANs. *$p<0.05$.

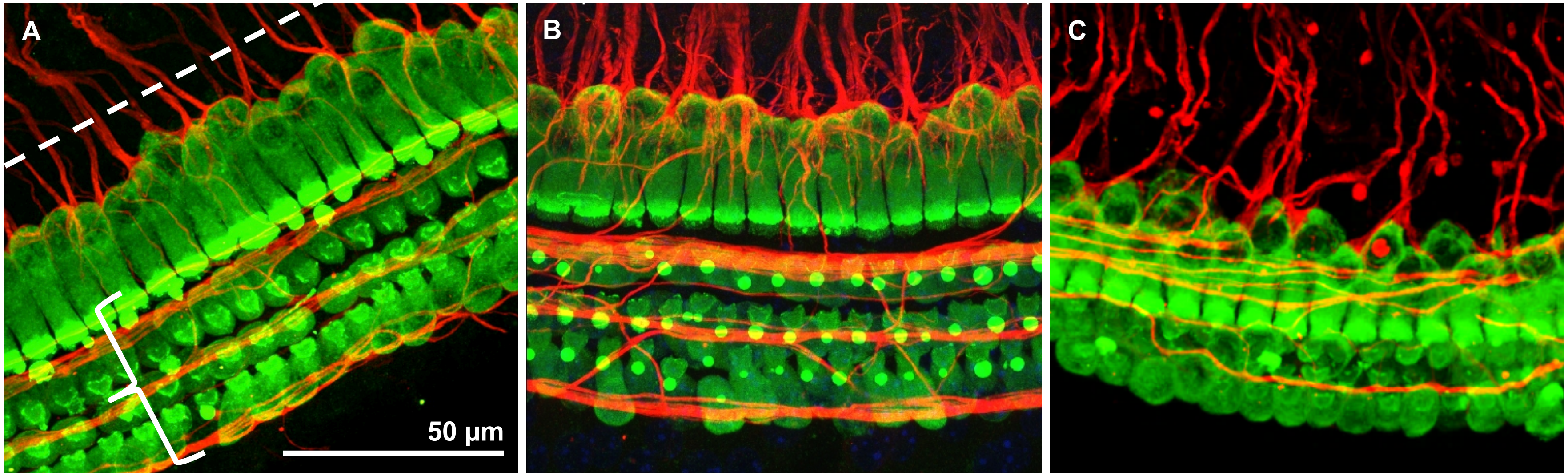

Supplementary Figure 2. Representative images of fibre disorganization showing a severity of **(A)** 0, in which neurites are intact; **(B)** 1, in which neurites are disorganized along the inner and outer hair cells; and **(C)** 2, in which neurites are severely disorganized and one cannot associate a given neurite with a hair cell. Dotted line in **A** shows where fibre disorganization was assessed for afferent fibres along IHCs and white bracket shows where fibre disorganization was assessed for three rows of efferent fibres along OHCs.
